# Estimating the effectiveness of a hospital’s interventions in India: impact of the choice of disability weights

**DOI:** 10.2471/BLT.14.147900

**Published:** 2015-05-15

**Authors:** Susmita Chatterjee, Richard A Gosselin

**Affiliations:** a Public Health Foundation of India, Plot No. 47, Sector 44, Institutional Area, Gurgaon, 122002, Haryana, India.; b Institute for Global Orthopaedics and Traumatology, University of California San Francisco, San Francisco, United States of America.

## Abstract

**Objective:**

To calculate the effect of using two different sets of disability weights for estimates of disability-adjusted life-years (DALYs) averted by interventions delivered in one hospital in India.

**Methods:**

DALYs averted by surgical and non-surgical interventions were estimated for 3445 patients who were admitted to a 106-bed private hospital in a semi-urban area of northern India in 2012–2013. Disability weights were taken from global burden of disease (GBD) studies. We used the GBD 1990 disability weights and then repeated all of our calculations using the corresponding GBD 2010 weights. DALYs averted were estimated for surgical and non-surgical interventions using disability weight, risk of death and/or disability, and effectiveness of treatment.

**Findings:**

The disability weights assigned in the GBD 1990 study to the sequelae of conditions such as cataract, cancer and injuries were substantially different to those assigned in the GBD 2010 study. These differences in weights led to large differences in estimates of DALYs averted. For all surgical interventions delivered to this patient cohort, 11 517 DALYs were averted if we used the GDB 1990 weights and 9401 DALYs were averted if we used the GDB 2010 disability weights. For non-surgical interventions 5168 DALYs were averted using the GDB 1990 disability weights and 5537 DALYS were averted using the GDB 2010 disability weights.

**Conclusion:**

Estimates of the effectiveness of hospital interventions depend upon the disability weighting used. Researchers and resource allocators need to be very cautious when comparing results from studies that have used different sets of disability weights.

## Résumé

**Objectif:**

Calculer l'impact de l'utilisation de deux séries différentes de coefficients de pondération pour le calcul des années d’incapacité sur les estimations relatives aux années de vie ajustées sur l'incapacité (DALY) évitées par des interventions pratiquées dans un hôpital indien.

**Méthodes:**

L'estimation des DALY évitées par des interventions chirurgicales et non chirurgicales a reposé sur 3445 patients admis en 2012-2013 dans un hôpital privé de 106 lits situé dans une zone semi-urbaine du nord de l'Inde. Les coefficients de pondération servant au calcul des années d’incapacité ont été tirés des études sur la charge mondiale de morbidité (CMM). Nous avons utilisé les coefficients de pondération de l'étude sur la CMM de 1990, puis refait tous nos calculs avec les coefficients de pondération de l'étude sur la CMM de 2010. L'estimation des DALY évitées grâce à des interventions chirurgicales et non chirurgicales s'est basée sur les coefficients de pondération servant au calcul des années d’incapacité, le risque de décès et/ou de handicap et l'efficacité du traitement.

**Résultats:**

Les coefficients de pondération appliqués dans l'étude sur la CMM de 1990 aux séquelles de maladies telles que la cataracte, le cancer et les traumatismes étaient nettement différents de ceux appliqués dans l'étude sur la CMM de 2010. Ces différences de coefficients de pondération ont entraîné des différences notables dans l'estimation des DALY évitées. En ce qui concerne l'ensemble des interventions chirurgicales subies par cette cohorte de patients, le nombre de DALY évitées s'élève à 11517 si l'on applique les coefficients de pondération de l'étude sur la CMM de 1990 contre 9401 si l'on applique ceux de l'étude sur la CMM de 2010. En ce qui concerne les interventions non chirurgicales, le nombre de DALY évitées s'élève à 5168 si l'on applique les coefficients de pondération de l'étude sur la CMM de 1990 contre 5537 si l'on applique ceux de l'étude sur la CMM de 2010.

**Conclusion:**

L'évaluation de l'efficacité des interventions hospitalières dépend des coefficients de pondération utilisés pour le calcul des années d’incapacité. Les chercheurs et les responsables de l'affectation des ressources doivent faire preuve de prudence lorsqu'ils comparent les résultats d'études dans lesquelles différentes séries de coefficients de pondération ont été appliquées pour calculer les années d’incapacité.

## Resumen

**Objetivo:**

Calcular el efecto del uso de dos grupos diferentes de pesos de discapacidad para estimaciones de años de vida ajustados en función de la discapacidad (AVAD) evitados por intervenciones llevadas a cabo en un hospital de la India.

**Métodos:**

los AVAD evitados por intervenciones quirúrgicas y no quirúrgicas se estimaron para 3445 pacientes que fueron ingresados en un hospital privado de 106 camas de un área semiurbana del norte de la India en 2012-2013.Los pesos de discapacidad se tomaron de estudios de carga mundial de morbilidad. Se utilizaron los pesos de discapacidad de 1990 de la carga mundial de morbilidad y a continuación se repitieron todos los cálculos utilizando los pesos de 2010 correspondientes. Los AVAD evitados se estimaron para intervenciones quirúrgicas y no quirúrgicas utilizando el peso de discapacidad, el riesgo de muerte o discapacidad y la eficacia del tratamiento.

**Resultados:**

Los pesos de discapacidad asignados en el estudio de 1990 de carga mundial de morbilidad a las secuelas de enfermedades como cataratas, cáncer y lesiones eran sustancialmente diferentes a los asignados en el estudio de 2010. Estas diferencias en los pesos provocaron grandes diferencias en las estimaciones de los AVAD evitados. Para todas las intervenciones quirúrgicas realizadas a esta cohorte de pacientes, se evitaron 11517 AVAD si usamos los pesos de discapacidad de 1990 de carga mundial de morbilidad y 9401 si usamos los de 2010. Para las intervenciones no quirúrgicas, se evitaron 5168 AVAD si se utilizan los pesos de discapacidad de 1990 de carga mundial de morbilidad y 5537 si se utilizan los pesos de 2010.

**Conclusión:**

Las estimaciones de la eficacia de las intervenciones hospitalarias dependen del peso de discapacidad utilizado. Los investigadores y los asignadores de recursos deben ser muy cautos al comparar los resultados de los estudios que han utilizado diferentes grupos de pesos de discapacidad.

## ملخص

**الغرض:**

حساب تأثير استخدام مجموعتين مختلفتين من أوزان العجز لتقديرات سنوات العمر المصححة باحتساب مدد العجز (DALYs) التي تم تفاديها بواسطة التدخلات المقدمة في مستشفى واحد في الهند.

**الطريقة:**

تم تقدير سنوات العمر المصححة باحتساب مدد العجز (DALYs) التي تم تفاديها بتدخلات جراحية وتدخلات غير جراحية لـ 3445 **  **مريضًا تم إدخالهم إلى مستشفى بسعة 106 **  **أسرّة في منطقة شبه حضرية في شمال الهند في الفترة من عام **  **2012 إلى عام **  **2013. ولقد تم أخذ أوزان العجز من دراسات العبء العالمي للمرض (GBD). لقد استخدمنا أوزان العجز للعبء العالمي للمرض في عام **  **1990 ثم قمنا بتكرار جميع حساباتنا باستخدام ما يقابلها من أوزان العبء العالمي للمرض في عام **  **2010. وقد تم تقدير سنوات العمر المصححة باحتساب مدد العجز (DALYs) التي تم تفاديها للتدخلات الجراحية والتدخلات غير الجراحية باستخدام أوزان العجز، ومخاطر الوفاة و/أو العجز، وفاعلية العلاج.

**النتائج:**

كانت أوزان العجز المخصصة في دراسة العبء العالمي للمرض في عام 1990 لعقابيل الحالات المرضية مثل إعتام عدسة العين والسرطان والإصابات مختلفة إلى حد كبير بالمقارنة مع تلك الأوزان المخصصة في دراسة العبء العالمي للمرض في عام 2010. وقد أدت هذه الاختلافات في الأوزان إلى وجود اختلافات كبيرة في تقديرات سنوات العمر المصححة باحتساب مدد العجز (DALYs) التي تم تفاديها. وبالنسبة لجميع التدخلات الجراحية المقدمة إلى هذه المجموعة من المرضى، فقد تم تفادي 11517 حالة من سنوات العمر المصححة باحتساب مدد العجز إذا استخدمنا أوزان العبء العالمي للمرض في عام 1990، كما تم تفادي 9401 حالة من سنوات العمر المصححة باحتساب مدد العجز إذا استخدمنا أوزان العجز للعبء العالمي للمرض في عام 2010. وبالنسبة للتدخلات غير الجراحية، فقد تم تفادي 5168 من سنوات العمر المصححة باحتساب مدد العجز باستخدام أوزان العجز للعبء العالمي للمرض في عام 1990 وتم تفادي 5537 من سنوات العمر المصححة باحتساب مدد العجز باستخدام أوزان العجز للعبء العالمي للمرض في عام 2010.

**الاستنتاج:**

تعتمد تقديرات فاعلية تدخلات المستشفى على أوزان العجز المستخدمة. ينبغي أن يكون الباحثون ومحصصو الموارد حذرين جدًا عند مقارنة نتائج الدراسات التي استخدمت مجموعات مختلفة من أوزان العجز.

## 摘要

**目的:**

旨在针对一家印度医院采取干预措施而避免的残疾调整生命年 (DALY) 的估测，核算出采用两组不同的残疾权重所产生的影响。

**方法:**

通过外科和非外科干预措施而避免的残疾调整生命年 (DALY) 是针对 3445 名患者做出的估测，而这些患者均在 2012-2013 年间收治于印度北部近郊地区一家设有 106 个病床的私立医院。残疾权重取自全球疾病负担 (GBD) 研究。 我们采用了 1990 年 GBD 的残疾权重，然后采用 2010 年 GBD 的相应权重重复进行我们所有的计算。 所避免的残疾调整生命年 (DALY) 是通过采用残疾权重、死亡和/或残疾风险以及治疗的有效性而针对外科和非外科干预措施做出的估测。

**结果:**

1990 年 GBD 研究中为白内障、癌症和伤病等疾病后遗症而分配的残疾权重与 2010 年 GBD 研究中分配的大不相同。 这些权重方面的差别让对所避免的残疾调整生命年 (DALY) 的估测产生了巨大差异。 关于提供至该种患者队列的所有外科干预措施，如果我们采用 1990 年的 GDB 权重，则避免了 11517 例残疾调整生命年 (DALY)；如果我们采用 2010 年的 GDB 残疾权重，则避免了 9401 例残疾调整生命年 (DALY)。 关于非外科干预措施，我们采用 1990 年的 GDB 残疾权重时避免了 5168 例残疾调整生命年 (DALY)，而在采用 2010 年的 GDB 残疾权重时则避免了 5537 例残疾调整生命年 (DALY)。

**结论:**

对医院所采取干预措施之有效性的估测取决于所采用的残疾权重。 在对比采用的多组不同残疾权重的研究结果时，研究人员和资源分配人员需要非常谨慎。

## Резюме

**Цель:**

Рассчитать эффект от использования двух различных наборов весовых коэффициентов для оценки количества лет жизни, потерю которых в связи с инвалидностью удалось предотвратить в результате мероприятий, проводимых в одной из больниц Индии.

**Методы:**

В частной больнице на 106 мест в полугородской зоне в северной Индии в 2012–2013 гг. была проведена оценка скорректированного с поправкой на инвалидность количества лет жизни для 3445 пациентов, потерю которых удалось предотвратить посредством хирургических и нехирургических вмешательств (спасенных лет жизни). Весовые коэффициенты были взяты из исследований глобального бремени заболеваний (ГБЗ). Сначала использовались весовые коэффициенты по инвалидности из исследований ГБЗ за 1990 год, а затем все расчеты были повторены с использованием аналогичных коэффициентов из исследований ГБЗ за 2010 год. Количество спасенных лет жизни оценивалось для хирургических и нехирургических вмешательств с использованием весовых коэффициентов по инвалидности, риску летального исхода и (или) инвалидности, а также по эффективности лечения.

**Результаты:**

Весовые коэффициенты по инвалидности, присвоенные в исследованиях ГБЗ от 1990 г. последствиям таких заболеваний и состояний, как катаракта, рак или травмы, значительно отличались от значений, присвоенных им в исследовании ГБЗ от 2010 г. Такое различие в весовых коэффициентах привело к значительной разнице при оценке количества спасенных лет жизни. Для всех случаев хирургических вмешательств, проведенных в отношении данной когорты пациентов, количество спасенных лет жизни составило 11 517, если пользоваться коэффициентами из исследования ГБЗ от 1990 г., и 9401 при использовании коэффициентов от 2010 г. Для нехирургических вмешательств количество спасенных лет жизни при использовании коэффициентов ГБЗ по инвалидности от 1990 г. составило 5168, а при использовании коэффициентов от 2010 г. — 5537.

**Вывод:**

Оценка эффективности больничных мероприятий зависит от используемых весовых коэффициентов по инвалидности. Исследователи и лица, отвечающие за выделение ресурсов, должны проявлять осмотрительность при сравнении результатов исследований, в которых использовались разные весовые коэффициенты.

## Introduction

Comprehensive summary measures of population health were estimated in the global burden of disease (GBD) 1990, 2004 and 2010 studies.^1^^–^^3^ The GBD 1990 study was commissioned by the World Bank and quantified the health effects of more than 100 diseases and injuries in each of eight regions of the world.^1^ The disability-adjusted life-year (DALY) was used to facilitate comparisons of health outcomes and measures of the effectiveness and cost–effectiveness of various interventions.^1^ Subsequently, there has been extensive debate on many of the variables that affect estimates of DALYs, such as the number of years lost on death, disability and age weights and time discounting.^4^^–^^9^

In the GBD 1990 study, an expert panel arbitrarily assigned disability weights to a comprehensive set of disease conditions, by using the so-called person trade-off method.^1^ After the results of the study were published, apparent inconsistencies in the derivation of these weights were noted.^10^

The GBD 2004 study,^2^ which focused mainly on injuries, was also criticized as the disability weights for several injuries appeared illogical.^10^ Such inconsistencies led to the appropriateness and usefulness of many disability weights being questioned.^10^ The GBD 2010 study^3^ tried to address these criticisms using multinational community and web-based surveys. In these surveys, more than 30 000 respondents were asked to choose the healthier of two hypothetical health states.^11^ Several researchers have pointed out that some of the disability weights estimated in the GBD 2010 study still do not make much sense.^10^^,^^12^

In spite of the numerous criticisms that the GBD team have tried to address, the DALY has been widely used by researchers, policy-makers and several other stakeholders since its inception. Here we estimate the DALYs averted for several surgical and non-surgical interventions among patients admitted to a hospital in India. We investigate the effect of using alternative disability weighting on the results.

## Methods

A 106-bed private hospital covering a semi-urban population in Uttar Pradesh, in northern India, was chosen for the study because its staff maintained a comprehensive computerized patient database and agreed to cooperate with the research team. As confidentiality issues prevented us from extracting data directly from the hospital’s paper-based records, we only extracted data from the computerized database. To calculate DALYs, we gathered data on each surgical admission to the hospital between 1 April 2012 and 31 March 2013. Because the hospital only began digitizing the records of non-surgical admissions at the start of 2013, we included patients admitted for a non-surgical intervention between 1 January 2013 and 31 March 2013. At the time of our study, the hospital did not keep records for outpatient and emergency services. We collected data on age, sex, length of stay, diagnosis and/or procedure for 3865 inpatients, which represented 43% of the 8936 patients who were admitted in the year beginning 1 April 2012. After excluding the 420 inpatients who had only been admitted for pain management or childbirth, we assigned disability weights to the remaining 3445 inpatients.

For each patient, we estimated the DALYs associated with conditions for which they were admitted and the DALYs averted by the surgical and non-surgical interventions that were carried out. First, we used the GBD 1990 disability weights and then repeated the analyses using the GBD 2010 weights. For injuries only, we did another set of calculations using the disability weights from the GBD 2004 study – which, with a few exceptions, were essentially based on the GBD 1990 weights.^2^ In each set of calculations we used identical scores for disease severity and the likelihood of treatment success.

We calculated DALYs averted using the method originally developed by McCord and Chowdhury^13^ but with slightly simplified estimates of the risks of death and disability and the effectiveness of treatment.^14^^–^^16^ Box 1 shows examples of our estimations of DALYs averted. These estimations were made without age weighting or discounting.

Box 1 **Examples of DALY-averted estimation**

- A 30-year-old female with appendicitis has a disease severity score of 1 (i.e. more than 95% chance of being fatal or disabling without surgery) and effectiveness-of-treatment score of 1 (i.e. more than 95% chance of being cured after surgery) with 54 years of life-to-live (life expectancy as per 2010 life table). A successful appendectomy will avert 54 × 1 × 1 × 0.326 = 18 DALYs using the 2010 disability weights.
- A one-year-old boy with septicaemia has more than 95% chance of death or disability without treatment and a chance of cure between 50% and 95% and 83.63 years of life-to-live. Successful medical treatment will avert 83.63 × 1 × 0.7 × 0.210 = 12 DALYs using the 2010 disability weights.

## Results

Specific disability weights were available in both the GBD 1990 and 2010 studies for 12 of the conditions for which our study inpatients were admitted (Table 1). For another 10 conditions, we were able to find a disability weight in the GBD 1990 study that appeared to be a potential match to one in the GBD 2010 study – or vice versa (Table 1).

**Table 1:** Disability weights assigned in the global burden of disease 1990 and 2010 studies

Condition	Disability weight		
	1990 study	2010 study	Difference^a^
**With matching conditions**			
Tuberculosis without HIV	0.274	0.331	−0.057
Severe diarrhoea	0.119	0.281	−0.162
Untreated terminal cancer	0.809	0.519	0.290
Infertility	0.180	0.011	0.169
Asthma	0.099^b^	0.132^c^	−0.033
Poisoning	0.611	0.171	0.440
Iodine deficiency goitre	0.025	0.200	−0.175
Chronic obstructive pulmonary disease	0.428^d^	0.383^e^	0.045
Femur fracture (treated)	0.272	0.072	0.200
Acute myocardial infarction	0.491	0.422^f^	0.069
Cirrhosis of liver	0.330	0.194	0.136
Benign prostatic hypertrophy	0.038	0.070	−0.032
**With nearly matching conditions**			
Cataract blindness	0.600	0.195^g^	0.405
Hydrocele	0.075	0.123^h^	−0.048
Ectopic pregnancy	0.549	0.326^i^	0.223
Appendicitis	0.463	0.326^i^	0.137
Lower respiratory infection	0.280	0.210^j^	0.070
Abscess	0.108^k^	0.005^k^	0.103
Phimosis	0.151^l^	0.123^h^	0.028
Hysterectomy	0.065^m^	0.225^n^	−0.160
Dengue fever	0.172	0.210^j^	−0.038
Chronic nephritic syndrome	0.104°	0.573^p^	−0.469

HIV: human immunodeficiency virus.

^a^ The 2010 study value subtracted from the 1990 study value.

^b^ Cases.

^c^ Uncontrolled.

^d^ Symptomatic cases.

^e^ Severe cases.

^f^ For days 1–2 post-infarction.

^g^ For distance vision – severe impairment.

^h^ For abdominopelvic problem – moderate.

^i^ For abdominopelvic problem – severe.

^j^ For infectious disease: acute episode, severe.

^k^ For open wound.

^l^ For stricture.

^m^ For postpartum haemorrhage.

^n^ Mean of values for “abdominopelvic problem – moderate” and “abdominopelvic problem – severe.”

^o^ For end-stage renal disease.

^p^ For end-stage renal disease; on dialysis.

Data source: the global burden of disease 1990 and 2010 studies.^1^^,^^3^

In the GBD 2010 study, disability weights for some surgical interventions differed markedly from those assigned in the GBD 1990 study. In consequence, our estimates of the total DALYs averted using GBD 1990 disability weights resulted in 11 517 DALYs, while using the GBD 2010 disability weights resulted in 9401 DALYs (Table 2). For example, our estimates of the numbers of DALYs averted by an abortion were 1649 when we used the disability weight given for abortion in the GBD 1990 study but 111 when we used the corresponding weight from the GBD 2010 study.

**Table 2:** Disability weights and disability-adjusted life-years averted for surgical interventions delivered in one hospital in India, April 2012–March 2013

Surgical condition	No. of cases	Disability weights			DALYs averted	
		1990 study	2010 study		1990 study	2010 study
Abortion	172	0.180	0.012		1649	111
Abscess	50	0.108	0.005		231	17
Anal fissure	82	0.108	0.005		59	8
Appendectomy	40	0.463	0.326		916	704
Caesarean section						
Elective	636	0.025	0.123		593	2957
Emergency	57	0.463	0.326		1406	1095
Calculus of kidney	25	0.107	0.123		105	134
Cataract	201	0.600	0.033		2599	156
Cholecystectomy	192	0.115	0.123		567	689
Circumcision	17	0.151	0.123		130	123
Dilation and curettage	278	0.065	0.012		874	164
Ectopic pregnancy	15	0.549	0.326		439	264
ERCP	44	0.115	0.123		110	125
Haematoma	55	0.065	0.225		133	468
Hernia repair	69	0.075	0.123		125	233
Hip replacement	16	0.108	0.171		32	60
Hydrocele	14	0.075	0.123		29	56
Hysterectomy	133	0.065	0.225		335	1177
Injury						
Crushing	7	0.218^a^	0.145		44	32
Face bones	10	0.223^a^	0.173		23	20
Femur	38	0.272^a^	0.072		124	42
Head	22	0.359	0.224		84	64
Patella, tibia and/or fibula	50	0.271^a^	0.070		277	75
Radius and/or ulna	14	0.180^a^	0.050		22	15
Scapula, clavicle and/or humerus	179	0.137	0.053		245	110
Other	28	0.074	0.080		77	73
Joint surgery	21	0.156	0.374		85	228
Mastectomy	24	0.086	0.038		70	34
Otitis media	14	0.023	0.018		5	4
Ovarian cyst	6	0.115	0.123		28	33
TURP	43	0.038	0.070		36	77
Wound debridement	28	0.108	0.005		28	1
Other surgery^b^	31	–	–		39	50
**Total**	**2611**	**–**	**–**		**11 517**	**9401**

DALYs: disability-adjusted life-years; ERCP: endoscopic retrograde cholangiopancreatography; TURP: transurethral resection of prostrate.

^a^ Same value as assigned in the global burden of disease 2004 study.^2^

^b^ Face laceration, suprapubic drainage, torsion testis and parotidectomy, which all have different disability weights and hence are not reported in table.

Note: Inconsistencies arise in some values due to rounding.

Data source: Disability weights are from the global burden of disease 1990 and 2010 studies.^1^^,^^3^

There were several conditions for which disability weights were not available in both the GBD 1990 and 2010 studies (e.g. hypertension). Further, in the GBD 2010 study, for example, no individual weights were given for peptic ulcer, kidney stone or appendicitis – although these conditions were loosely covered by the disability weights for abdominopelvic problems: mild, moderate or severe. Similarly, although the GBD 1990 study provided a specific disability weight for acute lower respiratory infection, no corresponding weight was included in the reported results of the GBD 2010 study. In our calculations based on the disability weights from the latter study, we used the weight given for infectious disease: acute episode, severe, as the weight for acute lower respiratory infection – assuming that all patients admitted for acute lower respiratory infection had a severe form of the infection.

Our estimates based on the GBD 1990 and GBD 2010 disability weights indicated that, over our study period, non-surgical interventions averted totalled 5168 and 5537 DALYs, respectively (Table 3). For a few non-surgical interventions, differences between the sets of disability weights that we used led to substantial differences in our estimates of the DALYs averted (Table 3). For example, our estimates of the numbers of DALYs averted by treating chronic nephritic syndrome with dialysis were 281 when we used the GBD 1990 disability weight but 1866 when we used the GBD 2010 weight.

**Table 3:** Disability weights and disability-adjusted life-years averted for non-surgical interventions delivered in one hospital in India, Jan 2013–March 2013

Diagnosis	No. of cases	Disability weights			DALYs averted	
		1990 study	2010 study		1990 study	2010 study
Acute lower respiratory infection	82	0.280	0.210		469	389
Chronic nephritic syndrome	158	0.104	0.573		281	1866
Chronic obstructive pulmonary disease	25	0.428	0.383		71	79
Dengue fever	15	0.172	0.210		43	57
Diabetes	42	0.078	0.099		26	44
Diarrhoea	51	0.086	0.281		68	231
Fever	34	0.172	0.053		0	0
Heart disease	38	0.227	0.167		154	140
Hepatitis	12	0.209	0.210		24	27
Hypothyroidism	12	0.025	0.200		9	83
Neonatal respiratory distress	164	0.323	0.186		3053	1883
Septicaemia	37	0.616	0.210		656	264
Tuberculosis	11	0.274	0.331		25	34
Typhoid fever	8	0.115	0.210		27	51
Urinary tract infection	34	0.107	0.210		0	0
Other non-surgical conditions^a^	111	–	–		260	389
**Total**	**834**	**–**	**–**		**5168**	**5537**

DALYs: disability-adjusted life-years.

^a^ Gastritis, pancreatitis, febrile convulsions, asthma, anaemia and pleural effusion, which all have different disability weights and hence not reported in table.

Note: Inconsistencies arise in some values due to rounding.

Data source: Disability weights are from the global burden of disease 1990 and 2010 studies.^1^^,^^3^

Our estimates based on the GBD 1990 disability weights indicated that, among the 3445 inpatients included in our analyses, total DALYs were 23 829. The corresponding value based on the GBD 2010 weights – 21 908 – was about 8% lower.

The GBD 2004 disability weights for fractures of the femur, radius or ulna, tibia and facial bones are the same as the corresponding GBD 1990 weights. For some procedures, however, the GBD 2004 disability weights were markedly different from those given in either the GBD 1990 study or the GBD 2010 study and these differences had an impact on our estimates of the DALYs averted by the procedures. For example, when we based our estimates on the disability weights assigned in the GBD 1990, 2004 and 2010 studies, it appeared that our study hospital had averted 245, 273 and 110 DALYs, respectively, by treating fractures of the clavicle, scapula and/or humerus. The corresponding estimates for treatment of intracranial injuries were 84, 86 and 64 DALYs averted, respectively.

## Discussion

We found that, for some conditions, our estimates of DALYs averted differed substantially according to which set of disability weights we used. It was not always possible to find perfect matches between the categories used in the GBD 1990 and 2010 studies. For example, cataract was given a GBD 1990 disability weight of 0.600 – under a cataract blindness category – but the most appropriate category in the GBD 2010 study appeared to be distance vision: moderate impairment, which had a much lower disability weight of 0.033. The GBD 2010 disability weights for more severe visual impairment, in the categories distance vision: severe impairment (0.191) or distance vision: blindness (0.195) were also much lower than the corresponding GBD 1990 values, as discussed elsewhere.^12^ Our estimates of the numbers of DALYs averted by abscess drainage, among 50 inpatients, were 231 when we used the GBD 1990 disability weights but only 17 when we used the GBD 2010 weights. For both of these estimates we had to use the disability weight for open wound – i.e. the most appropriate category that was common to the GBD 1990 and 2010 studies – while acknowledging that not all open wounds are drained abscesses. The GBD 1990 disability weight for open wound (0.108) was 22-fold higher than the corresponding GBD 2010 weight (0.005). Surgical treatment of anal fissure, wound debridement and some non-surgical conditions – e.g. diarrhoea, septicaemia, hypothyroidism and neonatal respiratory distress – also have GBD 2010 disability weights that were very different from their GBD 1990 equivalents.

The findings raise two important questions. First, which set of disability weights is most accurate? Second, does the best set of weights vary depending on the intervention or condition being investigated? As the method used to generate the GBD 1990 disability weights was completely different to that used to generate the GBD 2010 weights, it is perhaps not surprising that the two sets of weights show some differences. Although most studies on the cost–effectiveness of surgery and other conditions in low- and middle-income countries have used the GBD 1990 disability weights, future studies on the same topic are much more likely to use the GBD 2010 weights. As information on the cost of an intervention per DALY averted can be an important policy tool for resource allocation, researchers and resource allocators need to be very cautious when comparing results from studies that have used different sets of disability weights. Therefore, we are now evaluating whether the different sets of disability weights will affect the cost–effectiveness of the interventions available in the study hospital.

In the evaluation of disability weights, both the expert-panel approach of the GBD 1990 study and the survey approach of the GBD 2010 study led to some surprising and inconsistent results. We suspect that the respondents investigated in the GBD 2010 study were more biased towards acute pain and disability than to chronic impairment, and that some of them may have misunderstood what was meant by some of the conditions being investigated. The long-term impact of some interventions will vary substantially across countries. Leg amputation, for example, may impair function much less in settings where a prosthesis is available than in other settings. Although stratifying by geographical area or socioeconomic status might be preferable in theory, it would make the estimation process more complicated. In the design of a new set of disability weights, perhaps we should ask different questions and focus on the treatment required rather than the diagnosis. Is the disease or condition curable, treatable or only requiring palliation? Does it require medication only, minor surgery or major surgery? Does medication, if needed at all, need to be temporary or lifelong? Does the disease or condition affect cognition? Does it affect function or ability to work? After giving a severity weighting to the answers to these questions, a new measure of burden could be developed. However, we should keep in mind that DALYs were developed to measure disease burden not the burden of treatment. If future disability weights are based on surveys of lay people, they should be critically reviewed by experts to reduce inconsistencies.

Although we followed the same method to calculate DALYs as used by other researchers,^13^^–^^16^ our study has four major limitations. First, some of our inpatients’ admission diagnoses were not covered by specific GBD 1990 or GBD 2010 disability weights. For most of these diagnoses, we used the closest possible weights. Second, whenever there were separate disability weights for mild, moderate and severe forms of an admission diagnosis, we tended to be conservative and chose the weight for the moderate form. In the Indian context, mild cases are rarely admitted to hospital. Third, the digitized records of the study hospital often indicated a fracture as humerus/tibia without specifying whether the fracture was of the humerus, the tibia or both. Without access to radiographs and the patient’s charts, we had no way of distinguishing between arms and legs. In such cases, we were again conservative and used the disability weight for a fracture of the humerus – which, in both the GBD 1990 study and the GBD 2010 study, is lower than the disability weight for a fracture of the tibia. In consequence, our analyses included more fractures of the humerus than of the tibia – even though the latter are much more common in India. Whatever the scale of our misclassification bias, it remained unaltered by our choice of which set of disability weights to use. Finally, we had to assume that diagnoses were correct and that interventions were appropriate. Again, any related bias should not have been affected by our choice of which set of disability weights to use.

The evaluation of disability weights, which represent key components in the calculation of DALYs, remains very controversial. Though the GBD 2010 study attempted to respond to criticisms of the earlier GBD studies, many issues remain: the subjectivity in assigning disability weights to many given conditions, the many disability weights that make no medical sense, the non-inclusion of some conditions in the GBD studies and the difficulty in comparing studies that used different sets of disability weights. Perhaps some form of harmonization or consolidation of the GBD 1990 and GBD 2010 sets of disability weights should be considered. Although relatively few disability weights would require drastic adjustments, this would still lead to a third or, for some conditions, a fourth set of disability weights. While researchers, policy-makers and other stakeholders wait for the next set of disability weights, they need to keep in mind the limited comparability of studies based on the GBD 1990 disability weights and those based on the GBD 2010 weights.
